# Indication to Colectomy in the Hands of the Academic Gastroenterologist Versus the Surgeon: Old Giant Combat While Modern “Precision Medicine” Is Timidly Waving Hand to Offer Possible Help

**DOI:** 10.5152/tjg.2023.22567

**Published:** 2023-04-01

**Authors:** Giovanni Clemente Actis, Rinaldo Pellicano, Davide Giuseppe Ribaldone

**Affiliations:** 1 Independent Academic Gastroenterologist, Turin General Hospitals, Italy; 2 Department of Gastroenterology, Molinette-SGAS Hospital, Turin, Italy; 3 Department of Medical Sciences, University of Turin, Turin, Italy

Dear Editor,

We thank Dr. Evirgen for the reappraisal of the indications for intravenous cyclosporine (CsA) in binding down refractory ulcerative colitis (UC).^1^ Known initially as a plant anti-septic, its structure and immune suppressive mechanisms were unraveled early in the 1880s and then on “*it was just a ladder-climbing*” rush.

In the 1990s, our confident CsA-specific know-how intercepted the long-deceived expectations from the arena of immune disease, with its people frankly hating the death toll on rheumatism, psoriasis, and aggressive hepatitis patients. But the echoes of the very goal to be hit came in 1994 from the gastrointestinal unit of Mount Sinai Hospital, New York, where Lichtiger et al^2^ were enduring their combat against UC, an allegedly “autoimmune” inflammation of the gross intestine, known to force removal of the colon, as devastated by appallingly renovating bouts of inflammation over the years (a striking feature of UC).^2^ Luckily combining our efforts in Turin and New York, some significant evidence was shortly reached. (A) There was enough evidence accumulated to hold the fully autoimmune nature of UC; hence, its pathogenesis is driven by CsA-responsive T-lymphocytes; (B) To restrain those cells, 100 ng CsA/culture mL was the requirement; (C) This concentration had been shown to promptly follow a standard continuous CsA infusion (syringe pump venous catheter). Thus, at once, we had learnt how to really make CsA work and at which concentration. Present’s data of 1994 made the crucial kick in fostering our own infusion policies; we wanted to reduce the toxicities suspected to depend on excessive drug accumulation, and as a way to this goal, attention was devoted to the pharmacology of oral CsA. This approach eventually led to confirm the comparable toxicity and efficacy of the 2 formulations. As the due reward for those efforts, our originally elaborated infusion rate of 2 mg/kg is now universally accepted as the optimum standard.^3^ Lately, these figures were duplicated by colleagues working elbow-to-elbow at the kidney transplant program in our hospital.

We now must duly direct our attention onto the parallel existence at that time of a few projects based on similar starting points; these projects did bring about no deleterious competition, pressing rather onto momentarily set-aside viable ideas deserving revitalization. As the example above all, one may refer to the pioneering anti-tumor necrosis faxctor (TNF) preparations (infliximab) declared in August 2001 as the first monoclonal made available to treat Crohn’s disease.

The manipulation of the immune system with the specific issue of the treatment, and perhaps “the cure” of the immunological disorders as the feat to attain, has stimulated leading scientific centers since the 1950s. The original interest in the matters remained, however, pretty clinical, a specifically gloomy feeling being consistently spread by the dull news from several Mayo Liver Centers where a deceivingly short survival projection seemed for some time to play the black spell over the Mayo Centers. Rapidly grown in the USA since the 1970s, the Mayo Liver Centers really seemed to have materialized as a response to the epidemic-like cases of autoimmune hepatitis, and a rising plethora of disimmunities, that in those times battered relentlessly the Americans of Northern European descent. Things began to dramatically change when an intense algorithm of parenteral steroids was introduced.

Nowadays, limiting the discussion to the overly known hepatitis cases, the situation seems to present under 2 main aspects. Patients with autoimmune hepatitis in the case series seem to have behaved pretty well, with a life gain of many years. Over large populations, however, things are much less clear-cut, with an add-on of people suffering from progressive accumulation of drug toxicity (mostly overwhelming steroid damage) in the elderly patient subsets. Indeed, these words may not be totally reassuring, and a few further well-chosen considerations should help manage the trial patient crowd, and ourselves as not always at complete ease with our professions in the good clinical practice (GCP) world.

In the extreme synthesis, 2 main doubting chapters seem to have “tormented” trial drug designers, no matter where in the world.

(A) Trial mobs by definition may become recruited to include thousands of subjects all “lumped together” at once, because shown to share the trial requirements (limited to that enrolling occasion). In fact, however, the detection level common to average screening requirements can perhaps miss the next higher detectable level: as the unwanted chance then, genetically unrelated subjects could inadvertently be assigned the same treatment arm, placing in jeopardy the whole study value. Enough to spoil the sleep and career of any ambitious trialist; but a small research squad from the 4 corners of the world felt the need to reply to the request for assistance. These people made reference to the replication modes of a number of cell lineages exhaustively screened by them, reaching the following. Among many cell lineages, the progenitor cell might regularly possess the whole genomic and functional machinery, which can readily be recognized and attacked by an antibody response. The bulk of the lineage, however, is meant to mostly include a sort of second-class population, whereby these elements have maintained the mechanistic sequence of steps but have left behind the genetic organization marking the cell. Luckily, it was soon found that the antibody specificities raised by the contact with the second cell class were in fact sufficient to exert their constraining action, no matter if dealing with cells of the main lineage (first class) or the second line.^4^ In the ultimate analysis, this data promised to help mend at an acceptable cost the gaps bound to detract from the GCP’s traditional reliability.

(B) A few days apart, while still following the arguments described earlier, we almost stumbled into a paper dissecting the immune-regulating function of CXCL4 (a well-studied representative of the immune signaling molecules), taking a documented part in the management and containment of complex chain events, being well stimulatory, or down-grading. The present paper deals specifically with the chemokine CXCL4, probably an enhancer of scleroderma and lupus-like syndromes, according to the anticipations provisionally released by the multinational team led by senior author Franck Barrat.^5^ Further discussing matters with this expert, we agreed that, generically speaking, a wealth of chemokines may be found to interact with sequences of the cell synthetic machinery along the multiple processes of inflammation. However, to everyone’s surprise, CXCL4 exhibited the power to catalyze processes at a speed manifold that of known processes. Of the utmost interest, this chemokine was shown to be able to enjoy an express ride to the nucleus, sitting on the haughty back of its long-awaited ligand.

In conclusion, we deem it clear from the comments mentioned earlier that it is nobody’s intention to place under scrutiny the whole governing principles of GCP trials. However, an unduly exclusive interest level on the GCPs may obscure the perception from the trial assessment authority in the case of unique patient presentations, similar to the subject hereby described with the mutated chemokine.

In fact, all human beings have the equal right to be studied and helped, no matter if they come from a private office or a busy GCP.
